# LONGITUDINAL TRAJECTORIES OF EPIGENETIC CLOCKS IN HUMANS AND EFFECTS OF MEDICATION USE

**DOI:** 10.1093/geroni/igz038.129

**Published:** 2019-11-08

**Authors:** Sara Hagg

**Affiliations:** Karolinska Institutet, Stockholm, Sweden

## Abstract

We sought to investigate the longitudinal trajectories of the new generation epigenetic clock′s and how medication use is altering the DNA methylation age (DNAmAge). DNA methylation (Illumina 450k and EPIC) was assessed repeatedly up to six times (1992-2014) in whole blood (597 individuals, 1469 samples) from the Swedish Adoption/Twin Study of Aging (SATSA). DNAmAges were generated with the online calculator. Mean age at first measurement was 67 years (58% women). All clocks tested (Horvath, Hannum, Pheno, Grim, Skin&Blood) were correlated with chronological age (ρ=0.62-0.80). The steepest slope was found for Pheno while Horvath had the least steep slope. Correlations between the clocks ranged ρ=0.43-0.75. About 15% of the individuals started statin treatment during the follow-up, which changed the slopes to be less steep. Co-twin control analyses were confirmatory. Different DNAmAges are strongly correlated with each other in a longitudinal perspective. Treatment effects may alter the slopes of the DNAmAges.

